# Metabolic reprogramming of glycolysis and glutamine metabolism are key events in myofibroblast transition in systemic sclerosis pathogenesis

**DOI:** 10.1111/jcmm.16013

**Published:** 2020-11-02

**Authors:** John Henderson, Laura Duffy, Richard Stratton, Dianne Ford, Steven O’Reilly

**Affiliations:** ^1^ Faculty of Health and Life Sciences Northumbria University Newcastle Upon Tyne UK; ^2^ Centre for Rheumatic and Connective Tissue Diseases University College London London UK; ^3^ School of Biological and Biomedical Sciences Durham University Durham UK

**Keywords:** fibrosis, glutaminolysis, glycolysis, Systemic Sclerosis, TGF‐β1

## Abstract

Systemic Sclerosis (SSc) is a rare fibrotic autoimmune disorder for which no curative treatments currently exist. Metabolic remodelling has recently been implicated in other autoimmune diseases; however, its potential role in SSc has received little attention. Here, we aimed to determine whether changes to glycolysis and glutaminolysis are important features of skin fibrosis. TGF‐β1, the quintessential pro‐fibrotic stimulus, was used to activate fibrotic pathways in NHDFs in vitro. Dermal fibroblasts derived from lesions of SSc patients were also used for in vitro experiments. Parameters of glycolytic function were assessed using by measuring extracellular acidification in response to glycolytic activators/inhibitors, whilst markers of fibrosis were measured by Western blotting following the use of the glycolysis inhibitors 2‐dg and 3PO and the glutaminolysis inhibitor G968. Succinate was also measured after TGF‐β1 stimulation. Itaconate was added to SSc fibroblasts and collagen examined. TGF‐β1 up‐regulates glycolysis in dermal fibroblasts, and inhibition of glycolysis attenuates its pro‐fibrotic effects. Furthermore, inhibition of glutamine metabolism also reverses TGF‐β1‐induced fibrosis, whilst glutaminase expression is up‐regulated in dermal fibroblasts derived from SSc patient skin lesions, suggesting that enhanced glutamine metabolism is another aspect of the pro‐fibrotic metabolic phenotype in skin fibrosis. TGF‐β1 was also able to enhance succinate production, with increased succinate shown to be associated with increased collagen expression. Incubation of SSc cells with itaconate, an important metabolite, reduced collagen expression. TGF‐β1 activation of glycolysis is a key feature of the fibrotic phenotype induced by TGF‐B1 in skin cells, whilst increased glutaminolysis is also evident in SSc fibroblasts.

## 1 INTRODUCTION

Systemic Sclerosis (SSc) is characterized by dysregulated fibroblast to myofibroblast differentiation and excessive extracellular matrix deposition, resulting in skin fibrosis.^1^ The disease contains two subtypes: ‘limited’ (lSSc) and ‘diffuse’ (dSSc). The prognosis is significantly better for lSSc, with symptoms limited to skin thickening in discrete areas; however, for dSSc the skin symptoms are more severe and widespread, with fibrosis also occurring in internal organs which results in mortality.^2^ Although treatment for managing individual symptoms has improved, a pressing need for therapeutics that can significantly slow or even reverse the disease persists.^3^

Following the discovery of ‘the Warburg effect’,^4^ it is become well established that drastic alterations of the metabolic profile are a key feature of the majority of cancers. Although the enhanced glycolysis ubiquitous in cancer is well characterized, whether this occurs at the expense of the much more efficient energy producing OXPHOS pathway is controversial.^5^, ^6^ One hypothesis for this seemingly counter‐intuitive metabolic remodelling is glycolysis’ additional role in generating biosynthetic intermediates which support protein synthesis and cellular proliferation. Given that SSc is a disease in which myofibroblast proliferation and ECM synthesis are key, we sought to investigate whether metabolic changes are present and relevant to the pathogenesis. Recently, changes to the glycolytic phenotype have been identified in a number of other autoimmune^7^, ^8^, ^9^ and fibrotic disorders,^10^, ^11^ including TGF‐β‐mediated GLUT1 up‐regulation^12^—however, this is the first time metabolic reprogramming has been characterized in SSc.

In addition to glycolysis, glutamine metabolism is known to become significantly altered in cancer^13^, ^14^, ^15^ and is also becoming increasingly recognized as a potential mediator of pathogenicity in autoimmune^16^, ^17^ and fibrotic disorders.^18^ Glutamine metabolism is also similar to glycolysis in that it produces a number of substrates for biosynthetic pathways, thus supporting proliferation and protein synthesis. Particularly important in the context of fibrosis is the production of α‐ketoglutarate, an essential substituent of collagen I—the most abundantly expressed ECM component in fibrosis.

TGF‐β1 is a renowned activator of fibrotic genes and has been heavily implicated in the pathogenesis of SSc,^17^ making it a useful tool to model skin fibrosis in vitro. Using TGF‐β1 treated normal healthy dermal fibroblasts (NHDFs) and fibroblasts derived from SSc patient skin lesions, we show that TGF‐β1 enhances glycolytic parameters and that inhibition of both glycolysis and glutamine metabolism attenuates the expression of pro‐fibrotic markers. Furthermore, SSc patient‐derived fibroblasts with enhanced collagen I expression also displayed enhanced glycolysis, whilst glutaminase was up‐regulated in all SSc fibroblasts, regardless of enhanced collagen I. This frames glycolysis and glutamine metabolism as potentially key mediators of fibrosis in SSc that can be targeted therapeutically. We further demonstrate the pro‐fibrotic role of succinate and that the active metabolite itaconate reduced collagen in SSc fibroblasts.

## 2 MATERIALS AND METHODS

### 2.1 Ethics

Full ethical approval was given for this work by the Northumbria University ethics committee and the research ethics committee Sunderland under REC/13/NE/0089 and followed the declaration of Helsinki guidelines and full informed consent. Four SSc patients were included in the study. All four SSc patients were defined as early diffuse (2 years or less from first non‐Raynaud's symptom), and all patients were female mean age 54 years and positive for antibodies to scl‐70.

All SSc patients are on no treatment at all (treatment naïve patients). Healthy controls were derived from female donors undergoing adipose reduction surgery. In both patients and controls, a 5‐mm punch biopsy was taken from the affected area and dermal fibroblasts isolated from the biopsy by enzymatic digestion using dispase and used at passages 1‐10.

### 2.2 Reagents

All reagents used were purchased from Sigma‐Aldrich unless otherwise stated.

### 2.3 Cell culture

Dermal fibroblasts derived from early diffuse SSc patient lesions (n = 4) or healthy controls (n = 3) were grown in Dulbecco's Modified Eagle's Medium (DMEM, Gibco) supplemented with 10% foetal bovine serum (FBS, Gibco), 1% penicillin/streptomycin (Gibco), and 2 mmol/L L‐glutamine (Gibco) and kept at a temperature of 37°C in an atmosphere of 5% CO_2_. For TGF‐β1 experiments, cells were serum starved overnight prior to treatment with 10 ng/mL TGF‐β1 (ab50036, Abcam) for 48 hours in fresh media containing 0.1% FBS.

The inhibitors 2‐DG, 3PO and G968 (Sigma) were dissolved in dH_2_O (2‐DG) or DMSO (3PO and G968) and added at concentrations of 10 mmol/L, 8 and 10 µmol/L, respectively. DMSO concentrations were kept at 0.1% or below and constant for all wells. Cell viability for these doses was tested using trypan blue to ensure against cell toxicity. The concentration of the inhibitors was informed by the literature and verified by viability assays using alamar blue (Figure S1), whereby only doses at which no toxic effect occurred were chosen.

### 2.4 Western Blotting

After washing twice with 1× PBS, cells were lysed on ice using 1× RIPA buffer (10 mmol/L tris‐Cl pH 8.0, 0.1% sodium deoxycholate, 0.1% SDS, 1% Triton X‐100, 140 mmol/L NaCl) containing protease inhibitors. Protein lysates were denatured in laemmli buffer and then separated on a 10% SDS‐PAGE gel before being transferred onto a nitrocellulose membrane (Bio‐Rad). Immunoblotting was performed using the following primary antibodies diluted in 5% milk block: α‐tubulin (ab7291, Abcam), collagen I (ab138492, Abcam), hexokinase II (ab104836, Abcam), α‐SMA (Abcam, ab7817, Abcam), KGA/GAC (12855‐1‐AP, Proteintech). The secondary antibodies used were goat anti‐rabbit HRP (P0448, Dako) and rabbit anti‐mouse HRP (PO260, Dako), and the blot was developed using SuperSignal™ West Dura Extended Duration Substrate ECL detection reagent (Thermo), with the chemiluminescent signal exposed on a gel dock system (Syngene).

### 2.5 Metabolic assays

Glycolysis, glycolytic flux and glycolytic capacity were measured using a Seahorse XFp Analyser. On the day of the assay, cells were switched to Seahorse XF Base DMEM pH 7.4 (Agilent) supplemented with 2 mmol/L glutamine and any TGF‐β1 or treatment compounds in the media previously. The glycolytic parameters were then deduced by measuring the ECAR (extracellular acidification rate) in response to the sequential addition to the wells of glucose (10 mmol/L), oligomycin (1 µmol/L) and 2‐DG (50 mmol/L) (all Sigma). Oxidative phosphorylation was also assessed using the Seahorse Analyser by measuring the OCR (oxygen consumption rate) in cells incubated in Seahorse XF Base DMEM with 2 mmol/L glutamine‐ which is determined by the Seahorse analyser in real time in pmol/min of the media that surrounds the cells. This is then normalized by taking account of total protein (µg), and data are shown relative to control untreated as a percentage.

### 2.6 qRT‐PCR

SSc and HC dermal fibroblasts were isolated and cultured in DMEM as described above. RNA was isolated using Trizol (Invitrogen) and 1 µg of RNA was reverse transcribed using Superscript RT (Invitrogen). qPCR was performed using 2 µL cDNA with primers for Hexokinase 2 (HK2) for 5′ TTCTTGTCTCAG ATTGAGAGTGAC 3′, HK2 rev 5′ TTGCAGGATGGCTCGGACTTG 3′; GLUT1 for 5′ CTTCCAGTATGTGGAGCAACTGT, GLUT1 rev 5′ GCACAGTGAAGATGATGAAGACG 3′, PFKFB3 for 5′ ACCAAAGATCACCCACGGATGT 3′, PFKFB3 rev 5′ AGCGAGTGCAGAATGGACACAA 3′ and 18S: for 5′‐GAATGGCTCATTAAATCAGTTATGG‐3′, rev 5′‐TATTAGCTCTAGAATTACCACAGTTATCC‐3’ using SYBR green (Sigma). Data were normalized to the housekeeping gene 18S and relative expression quantified using the delta‐delta Ct method from four independent donors. All samples were run in triplicate and samples lacking RT were run as negative controls to confirm target amplification.

Fibroblasts were cultured with or without TGF‐β1 alone or pre‐treated for 1 hour with the Smad inhibitor SB431542 (Sigma) 1 µmol/L or DMSO vehicle control (0.1% v/v) and stimulated for 48 hours in culture. After 48 hours RNA was harvested and qPCR was performed with specific primers for GLS‐1 for 5’‐CTGGAAGCCTGCAAAGTAAAC‐3’, Rev 5’‐TGAGGTGTGTACTGGACTTGG‐3’. Data are normalized to 18S and shown as fold change compared to control. Fibroblasts were culture with SB431542 (Sigma) at 1 µmol/L or DMSO vehicle control (0.1% vol/vol) and then all cultures treated with TGF‐β1 (10 ng/mL) for a further 36 hours and then RNA isolated by Trizol and qRT‐PCR performed for collagen1A1 as previously described.^19^ Also cells were incubated with the Fibroblast Growth Factor Receptor 3 (FGFR3) inhibitor PD173074 (Sigma, UK) 20 nmol/L (dose was chosen based on previous study^20^) or DMSO vehicle control and then all cultures treated with TGF‐β1 (10 ng/mL) for a further 36 hours and then RNA isolated by Trizol and qRT‐PCR performed for Gls1 as described above.

### 2.7 NAD+ measurements

Following a 48‐hour incubation in TGF‐β1, cells were trypsinized and re‐suspended in 1× PBS. The cell suspension was then mixed with a 0.2 mol/L NaOH 1% DTAB solution before the addition of 0.4 mol/L HCl, and then incubated at 60°C for 15 minutes to lyse the cells under conditions in which NAD^+^ is stable and NADH is degraded. NAD^+^ levels were then quantified using a commercially available luminescent cycling assay (G9071, Promega) as detailed in the manufacturer instructions.

### 2.8 Succinate quantification

NHDFs were cultured with or without TGF‐β1 (10 ng/mL) for 36 hours after which time the cells were removed and succinate was quantified according to manufacturer's instructions colorimetrically (Abcam). Four individual donors were used and all ran in triplicate.

### 2.9 Succinate treatment

NHDFs were cultured in 6‐well plates and then treated with succinate (5 mmol/L‐ Sigma) or untreated in low FBS DMEM and after 48 hours the RNA was extracted and qPCR was performed for collagen1A1 and 18S. Data were normalized to 18S and was from 4 independent cultures. Primers for collagen1A1 forward 5′‐CAA GAG GAA GGC CAA GTC GAG G‐3′, reverse 5′‐ CGT TGT CGC AGA CGC AGA T‐3’. All samples were run in triplicate and samples lacking RT served as negative controls.

In other experiments, cells were treated with succinate (5 mmol/L) or untreated and after 15 minutes stimulation cells were lysed in RIPA buffer containing a cocktail of protease and phosphatase inhibitors (Thermo Fisher). The lysate was subjected to PAGE, and Western blotting was performed using phosphorylated P38 MAPK thy180‐ty182 residues 1:600 dilution (Cell signaling technology) and was reprobed with beta‐actin 1:20,000 dilution (Abcam).

### 2.10 Smad‐binding element reporter assay

SBE luciferase reporter that contains four copies of the Smad‐binding element was used and 500 ng was transfected into NDHFs using Lipofectamine 3000 (Thermo Fisher) in six‐well plates with *Renilla* used to normalize transfection efficacy. After 24 hours post‐transfection Succinate or nothing was added to the cells (5 mmol/L) and after a further 24 hours cells were lysed and luciferase was measured using a luminometer. All samples were run in duplicate and three independent samples were used in this experiment.

### 2.11 4‐Octyl Itaconate treatment

SSc dermal fibroblasts were cultures in 6‐well plates from three separate donors and treated with the cell permeable derivative of itaconate 4‐octyl itaconate (Sigma) at 100 µmol/L or DMSO as this is the vehicle. This concentration was chosen as this appeared to be effective in inhibiting IL‐1β and IL‐6 release in macrophages.^21^ After 48 hours the cells were lysed and subjected to Western blotting for collagen 1 as described above. The blot was reprobed for β‐actin (Abcam,UK) to confirm equal loading of total protein.

4‐octyl itaconate was administered to SSc fibroblasts or DMSO and after 24 hours RNA was harvested from the cells using Trizol and RT‐qPCR was performed for the nuclear factor erythroid 2‐related factor (nrf‐2) target gene HO‐1 with specific primers HO‐1 For 5’‐TATCGTGCTCGCAATGAACACTCTG ‐’ Rev 5’‐GTTGAGCAGGAAGGCGGTCTTAG‐3’ and 18S: for 5′‐GAATGGCTCATTAAATCAGTTATGG‐3′, rev 5′‐TATTAGCTCTAGAATTACCACAGTTATCC‐3’ using SYBR green (Sigma, UK). Data were normalized to the housekeeping gene 18S and relative expression quantified using the delta‐delta Ct method. All samples were run in triplicate and samples lacking RT were run as negative controls to confirm target amplification.

### 2.12 Statistical analysis

Data for the NAD^+^ assays are presented as the mean ± standard error of the mean (SEM) and a Student's *t* test was used to test for significance between the TGF‐β1 treated and untreated NHDFs. For the analysis of OXPHOS/glycolytic parameters, OCR/ECAR is expressed as the mean percentage of the control mean. A *t* test was used for the OXPHOS assay, whereas significance was probed using one‐way ANOVA for parameters of glycolytic function and a Bonferroni post hoc test was performed to test for significance between all the different treatment conditions. For all other analysis, a *t* test was performed. A value of *P* < .05 was set as the threshold of significance for all analyses performed.

## 3 RESULTS

### 3.1 TGF‐β1 enhances glycolysis in NHDFs

**FIGURE 1:**
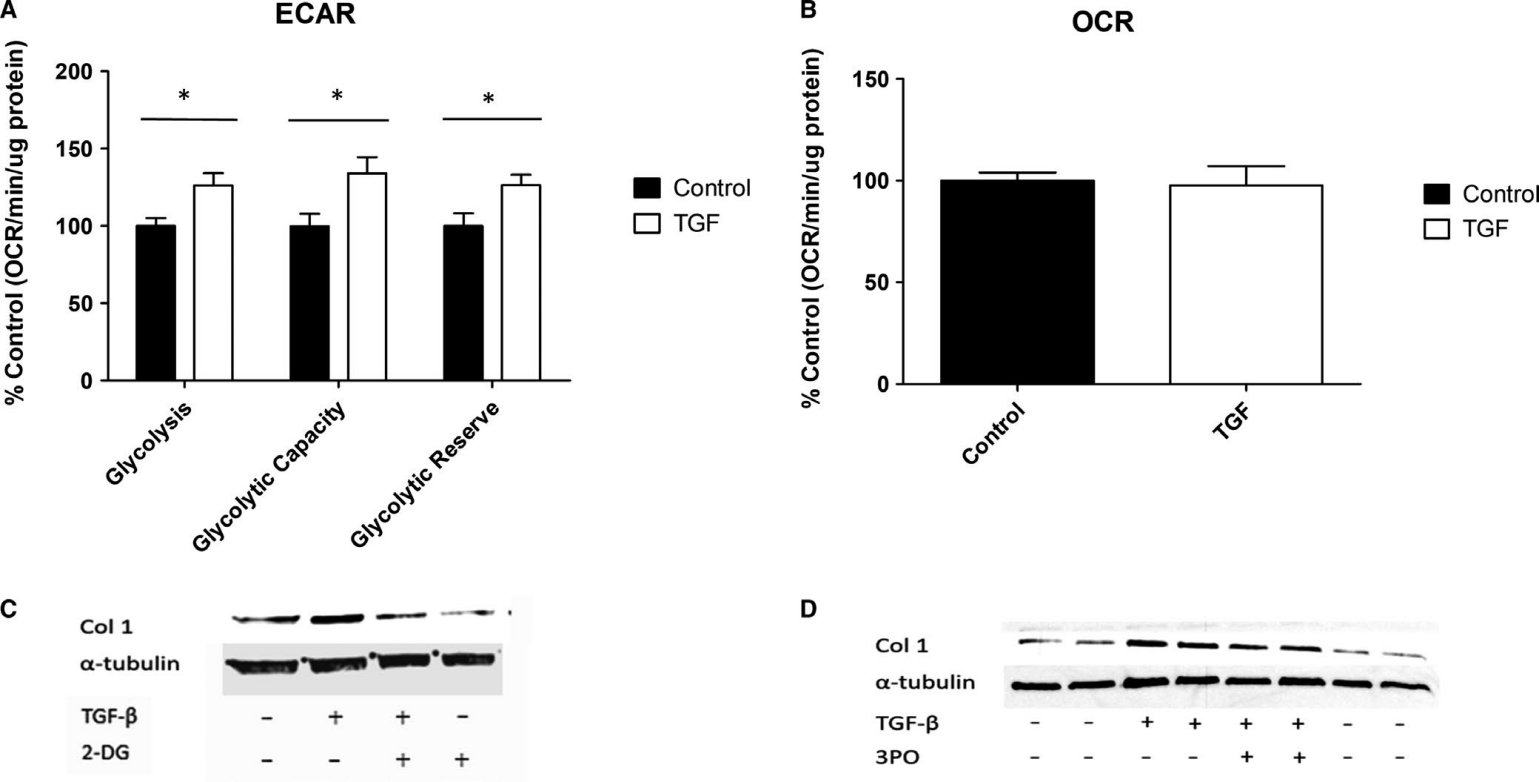
Glycolysis is a vital component of TGF‐β1 induction of fibrotic proteins in NHDFs. A, The glycolytic parameters of NHDFs treated with/without 10 ng/mL TGF‐β1 were measured on a Seahorse XFp Analyser by performing a glycolysis stress test. B, OXPHOS was also measured in NHDFs treated with/without 10 ng/mL TGF‐β1, by measuring the oxygen consumption rate (OCR). Data shown are the percentage change compared with control untreated cells, and OCR is measured by pmol/min normalized to total protein. C, NHDFs were treated with/without 10 ng/mL TGF‐β1 and/or the glycolysis inhibitor 2‐DG (10 Mm), and the proteins indicated were measured by Western blotting. D, Likewise, Western blotting was used to observe changes to proteins of interest in NHDFs were treated with/without 10 ng/mL TGF‐β1 and/or the glycolytic flux inhibitor 3PO (8 µmol/L). *Represents *P* < .05. Error bars represent the mean (n = 3) ±SEM. Statistical significance was tested for using 1‐way ANOVA followed by a Bonferroni post hoc test

To determine whether the pro‐fibrotic stimulus TGF‐β1 induces metabolic changes in NHDFs, basal OXPHOS and parameters of glycolytic function were measured following TGF‐β1 treatment. We found no notable changes to OXPHOS (Figure 1B), whereas there were large changes to glycolysis (Figure 1A), with basal glycolysis significantly enhanced (1.26‐fold, *P* = .02). In addition to basal glycolysis, the other glycolytic parameters measured were glycolytic capacity (glycolysis under conditions in which ATP generation via OXPHOS is inhibited, forcing glycolysis to work at maximal capacity) and glycolytic reserve (the difference between basal glycolysis and the glycolytic capacity), both of which were also up‐regulated (glycolytic capacity—1.34‐fold, *P* = .03, glycolytic reserve—1.26 fold, *P* = .04).

The increases in glycolytic capacity and reserve suggest that TGF‐β1 treated cells are still somewhat dependent on OXPHOS; however, they are much better equipped to deal with mitochondrial inhibition and quickly up‐regulate glycolysis as required. There was no significant difference apparent in the OCR in TGF‐β1 treated cells compared with control (Figure 1B—no significant difference between groups).

### 3.2 Inhibition of glycolysis attenuates TGF‐β1 induced fibrosis in NHDFs

Although there was up‐regulation of glycolysis following TGF‐β1 stimulation, given TGF‐β1 is a multi‐functional cytokine we sought to identify whether enhanced glycolysis was relevant to the pro‐fibrotic phenotype, using the prototypical glycolytic inhibitor 2‐deoxyglucose (2‐DG).^22^ 2‐DG potently reduces expression of the fibrotic marker collagen I following TGF‐β1 treatment (Figure 1C), although given 2‐DG completely blocks glycolysis this would be an unsuitable therapeutic for SSc treatment. However, the glycolytic flux inhibitor 3PO, which inhibits cellular capacity to quickly up‐regulate glycolysis rather than glycolysis per se,^23^ was also able to attenuate the TGF‐β1‐driven increase in collagen I (Figure 1D) and thus represents a feasible alternative to 2‐DG in the context of disease treatment.

**FIGURE 2:**
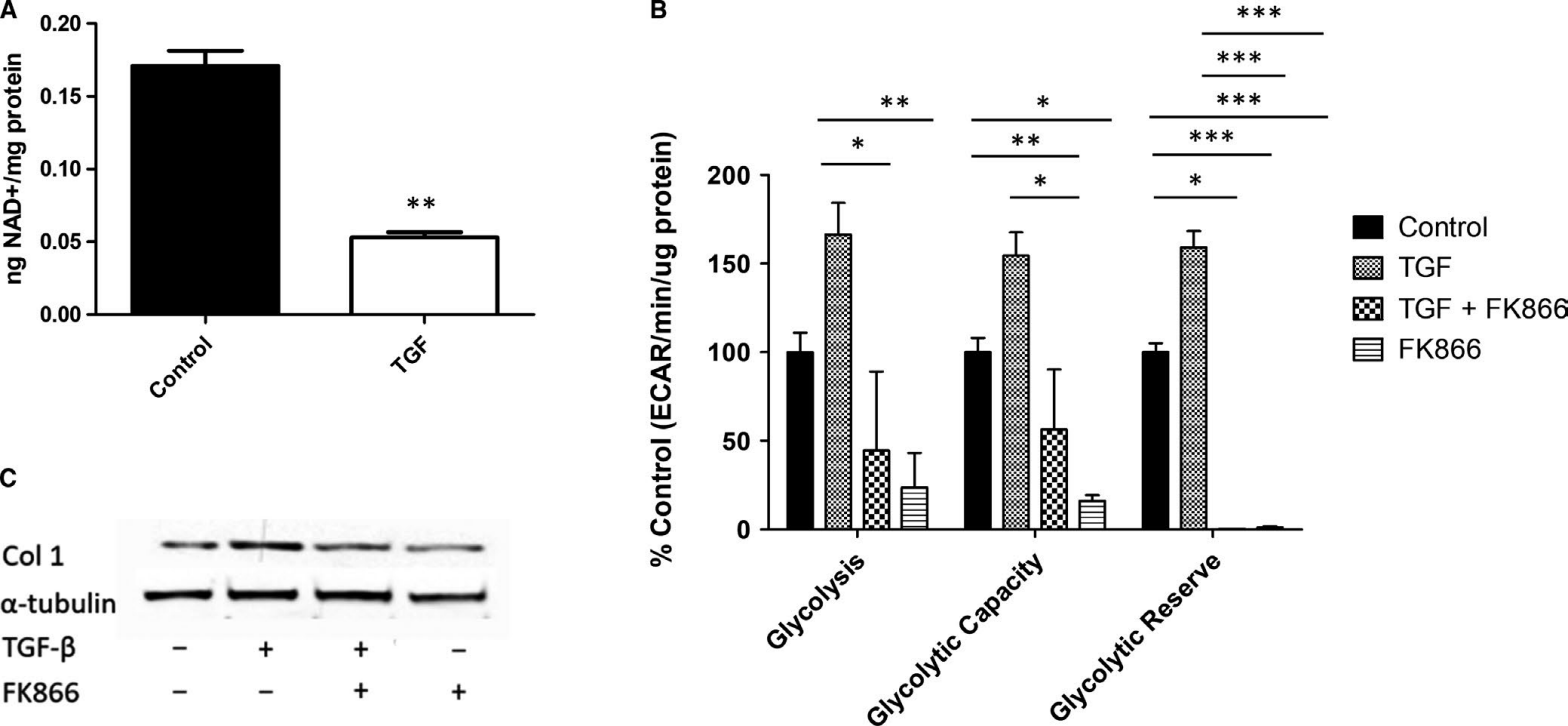
NAD^+^ levels regulate glycolysis and fibrotic expression in TGF‐β1 NHDFs. A, NAD^+^ levels were measured for NHDFs treated with/without 10 ng/mL TGF‐β1 using a commercial kit and normalized to protein levels. Statistical significance was tested for using a Student's *t* test and is highly significant. B, The glycolytic parameters of NHDFs treated with/without 10 ng/mL TGF‐β1 and or 10 nmol/L FK866 were measured on a Seahorse XFp Analyser by performing a glycolysis stress test. Statistical analysis was performed using 1‐way ANOVA and significant differences between the treatments tested using a Bonferroni post hoc test. C, Expression of collagen I and α‐tubulin was measured by Western blotting for NHDFs treated with/without 10 ng/mL TGF‐β1 and or 10 nmol/L FK866. *Represents *P* < .05, **P < .01, ***P < .0001. Error bars represent the mean (n = 5 for the NAD^+^ assay and n = 3 for the glycolysis stress test) ±SEM

Given that the conversion of NAD^+^ to NADH is a key step in glycolysis, NAD^+^ is a limiting factor for glycolytic activity and is depleted following TGF‐β1 up‐regulation of glycolysis (3.2‐fold, *P* = .008) (Figure 2A). Because of this dependency on NAD^+^ availability, diminishing the cellular NAD^+^ reserve by inhibiting nicotinamide phosphoribosyltransferase (NAMPT) (the rate‐limiting enzyme in NAD^+^ production via the NAD^+^ salvage pathway) with the specific inhibitor FK866 causes a significant decrease in glycolysis (TGF‐β1 vs TGF‐β1 + FK866 3.7‐fold, *P* = .0043), glycolytic capacity (TGF‐β1 vs TGF‐β1 + FK866 2.74‐fold, *P* = .0005) and glycolytic reserve (TGF‐β1 vs TGF‐β1 + FK866 455‐fold, *P* < .0001) (Figure 2B). This also translates to reduced collagen production in TGF‐β1 treated NHDFs (Figure 2C), once again highlighting the important role of glycolysis for the induction of fibrosis in vitro. FK866 is anti‐cancer agent which can be cytotoxic to cells; hence, prior to these experiments a dose‐response curve using alamar blue to assess viability was done which identified 10 nmol/L as a non‐toxic dose.

### 3.3 Inhibition of glutamine metabolism antagonises TGF‐β1 enhancement of glycolysis and fibrosis in NHDFs

Glutamine metabolism, aka glutaminolysis, is another metabolic pathway, which plays a key role fuelling cellular growth and thus may also contribute to the myofibroblast transition. Glutaminolysis yields the key ECM precursor α‐ketoglutarate as well as providing nitrogen required for pyridine and purine biosynthesis—an important pathway to facilitate enhanced proliferation.

**FIGURE 3:**
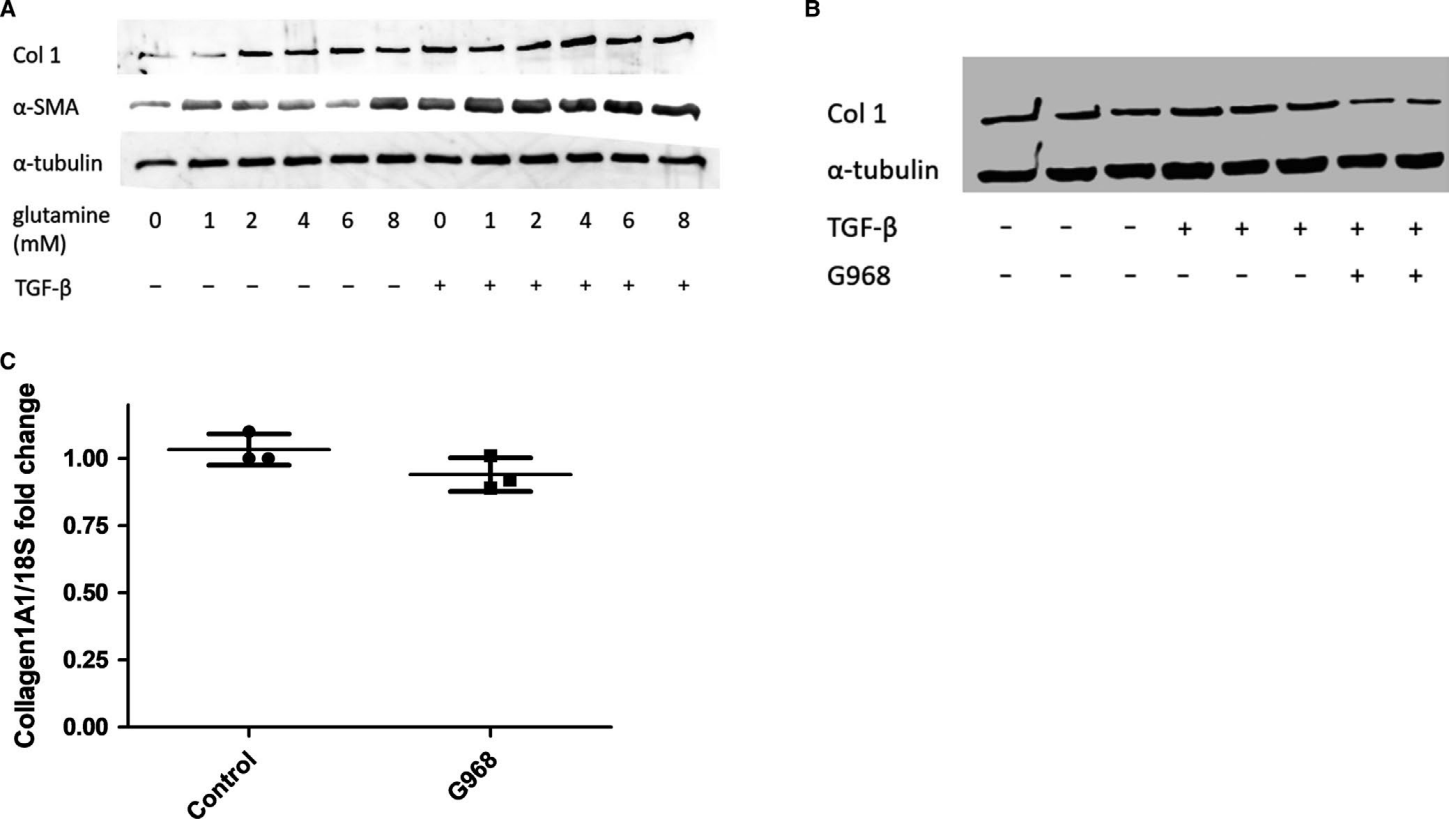
Glutaminase activity is essential for TGF‐β1 driven effects. A, NHDFs were treated with/without 10 ng/mL TGF‐β1 and various concentrations of L‐glutamine. Proteins of interest were quantified by Western blotting. B, The expression of Collagen I and α‐tubulin was quantified by Western blotting for NHDFs treated with/without 10 ng/mL TGF‐β1 and/or the specific glutaminase inhibitor G968 (10 µmol/L). C, The glutaminase inhibitor did not change the expression of collagen1A1 gene expression. Cells were treated with or without the glutaminase inhibitor G968 (10 µmol/L) for 36 h after which qRT‐PCR was performed for collagen1A1 gene expression *P* = .13 Student's *t* test n = 3. Data are the mean and SD and is normalized to 18S as the housekeeping gene (n = 3)

To test the importance of glutamine to TGF‐β1 induced fibrosis, varying concentrations of glutamine were added to NHDFs treated with or without TGF‐β1, and the fibrotic markers collagen I and α‐SMA were measured (Figure 3A). Other than a surprising increase in α‐SMA production at 8 mmol/L glutamine for the cells without TGF‐β1 stimulation, the effect of varying glutamine concentration is unremarkable. However, this was contradicted by the suppression of collagen expression when TGF‐β1 treated cells were co‐incubated with the specific glutaminase inhibitor G968 (Figure 3B). However, the glutaminase inhibitor did not alter the gene expression of collagen1A1 in the presence of TGF‐β1 (Figure 3C) control 1.03 (SD 0.05) v 0.94 (SD 0.06) *P* = .131 no significant difference Student's *t* test n = 3. On the whole, this evidence suggests glutamine metabolism is important for TGF‐β1 mediated fibrosis, with the conflicting results from the glutamine titration possibly due to the cellular glutamine reserve not being fully depleted after removal of glutamine from the media.

### 3.4 Glutamine metabolism but not glycolysis is ubiquitously up‐regulated in SSc fibroblasts

**FIGURE 4:**
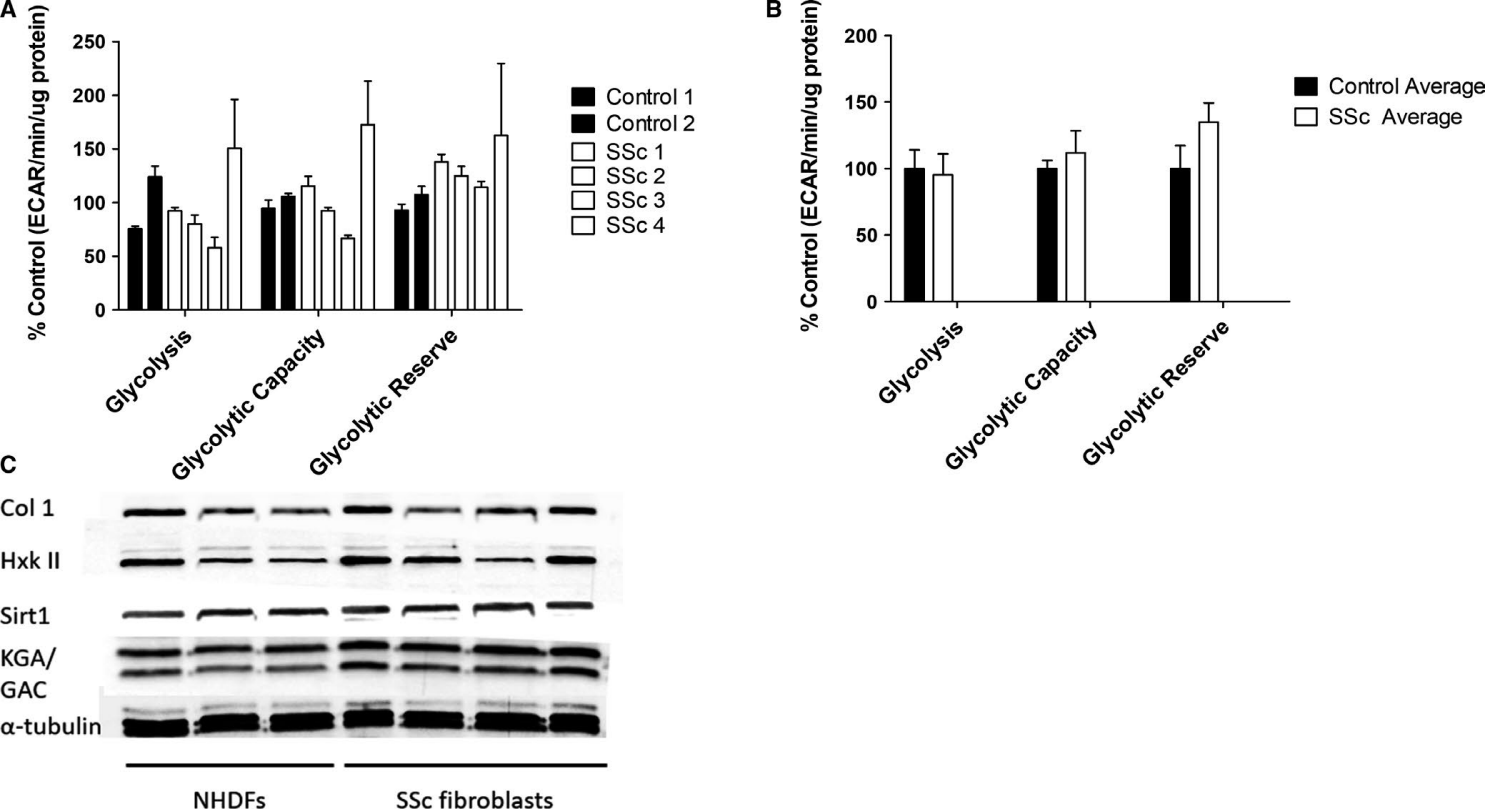
Glycolysis and glutaminase expression in SSc patient derived dermal fibroblasts. Glycolytic parameters for dermal fibroblasts from healthy controls (n = 3) and SSc patients (n = 4) were measured using a glycolysis stress test, displayed individually (A) and as disease and control averages (B). Statistical analysis was performed using 1‐way ANOVA. C, Expression of glutaminase (KGA/GAC), hexokinase II, collagen I and α‐tubulin in SSc and healthy control fibroblasts was analysed by Western blotting. Error bars represent the mean (n = 3) ±SEM

Glycolysis was measured in SSc fibroblasts derived from patient skin lesions (n = 4) and compared with healthy controls (n = 3) (Figure 4A). There was large variation within the cohort, perhaps reflecting the heterogeneous nature of the disease. SSc fibroblasts from only one donor showed an increase (non‐significant) in glycolysis compared with controls, whilst glycolysis was significantly down in another patient's fibroblasts. Interestingly, despite missing out on statistical significance the glycolytic reserve was increased in the fibroblasts from all patients (1.34‐fold average increase) suggesting these cells may have an enhanced capacity to become glycolytic under conditions of impaired OXPHOS. It is also noteworthy that collagen I expression was only up‐regulated in the two sets of SSc fibroblasts with both the highest glycolytic reserve (increased by 1.38‐fold and 1.57‐fold respectively) (Figure 4A,B) and expression of the glycolytic enzyme hexokinase II, whereas it was down in those with reduced glycolysis (Figure 4C). Contrastingly, all of the SSc fibroblasts showed an increase in glutaminase expression (Figure 4C), suggesting that unlike glycolytic changes, altered glutamine metabolism may be a ubiquitous trait in SSc. There was found two SSc patient samples elevated HK2 (Figure 4C). qRT‐PCR analysis showed that, the rate‐limiting enzyme HK2 and the glucose transporter GLUT1 were significantly elevated in SSc fibroblasts compared with healthy controls cells, but that PFK3 was not up‐regulated (Table 1).

**TABLE 1:** Gene expression of glycolytic genes in healthy control and SSc fibroblasts

Gene	Fold change SSc versus control (SD)	Significant (Student's *t* test)
HK2	2.7 (0.37)	Yes *P* = .0001
GLUT1	3.4 (1.44)	Yes *P* = .01
PFK3	1.14 (0.22)	No *P* = .26

### 3.5 TGF‐β1 up‐regulates glutaminase‐1 via TGF‐β receptor/Smad

**FIGURE 5:**
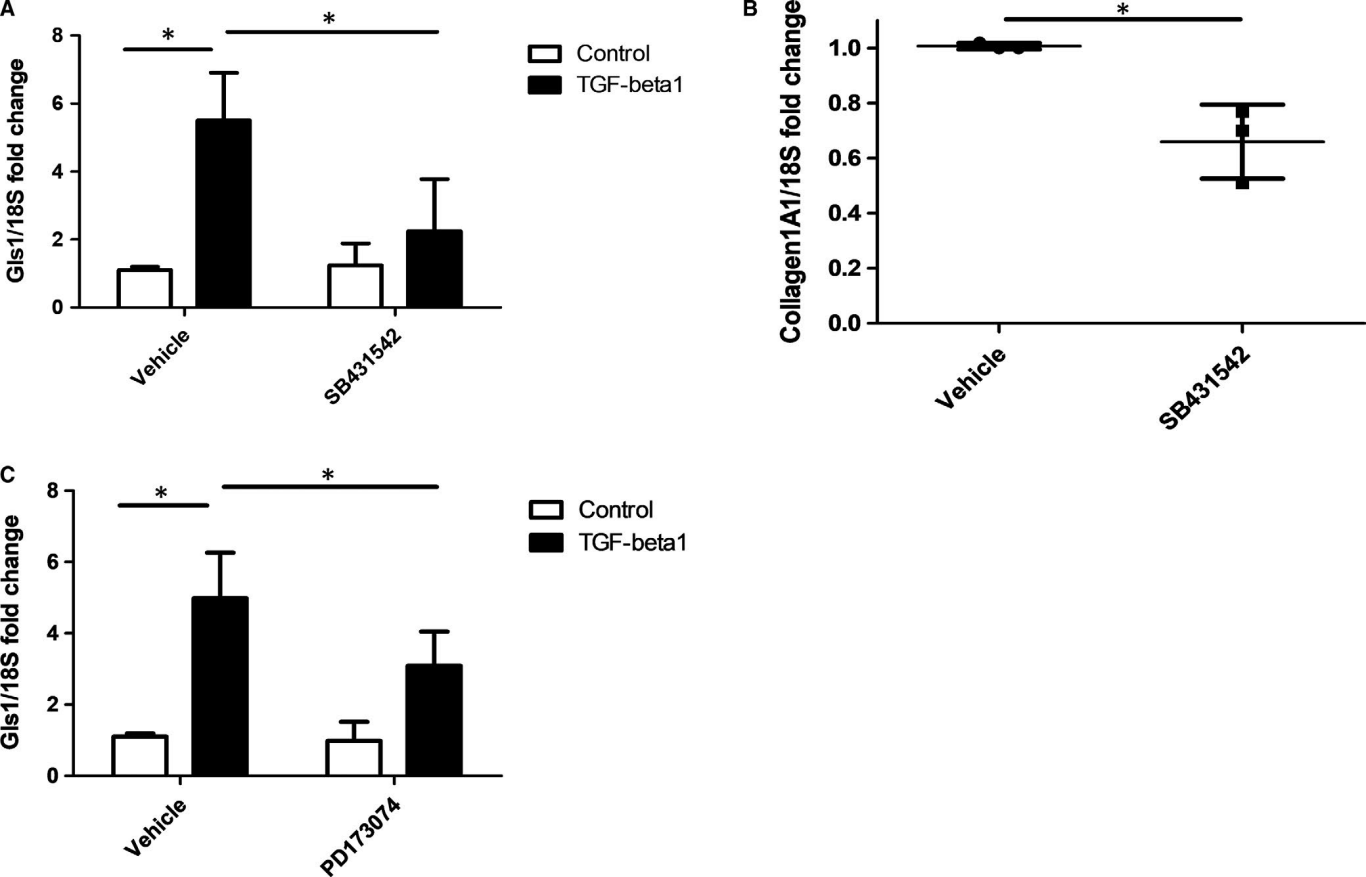
TGF‐β1 up‐regulates Gls1 in dermal fibroblasts via Smad. A, Expression of Gls1 was measured by qRT‐PCR after stimulation with TGF‐β1 and pre‐treated with the Smad inhibitor SB431542 (1 µmol/L) or vehicle control. Data are the mean and standard deviation *= Significant Two‐way ANOVA; n = 4 donors. B, Collagen1A1 gene expression was quantified after vehicle control or SB431542 and after 48 h with TGF‐β1 incubation. Data were normalized to the housekeeping gene 18S and shown as fold change compared with vehicle control treated cells. Data are the mean and SD *= significant Student's *t* test *P* = .0113; n = 3 donors. C, Expression of Gls1 was measured by qRT‐PCR after stimulation with TGF‐β1 and pre‐treated with the FGFR3 inhibitor PD173074 (20 nmol/L) or DMSO vehicle control treated. Data are the mean and standard deviation. *=Significant Two‐way ANOVOA; n = 4

Given that the glutaminase inhibitor G968 potently reduced TGF‐β1 induction of collagen, we sought to identify if TGF‐β‐1 could lead to induction of the enzyme that mediates glutaminolysis, GLS1, in stimulated fibroblasts. Figure 5 shows that incubation of normal healthy fibroblast with TGF‐β1 led to a significant up‐regulation of GLS1 mRNA and that this could be reduced, at least partially, through Smad inhibition (5.5 fold change TGF‐β1 and vehicle vs 2.25 TGF‐β1 and SB431542 *P* = .003; n = 4). Also, cells treated with Smad inhibitor also reduced collagen at the mRNA level *P* = .0113 Student's *t* test (Figure 5B) Vehicle treated fibroblast 1 vs SB431542 0.66 (0.01; 0.13 SD). To probe for other pathways outside of Smad, we used the FGFR3 inhibitor PD173074 to inhibit this receptor incubation of dermal fibroblast with PD173074 (20 nmol/L) suppressed TGF‐β1‐mediated increase in GLS1 expression, indicating that FGFR3 is partly mediating this effect *P* = .004 n = 4 (Figure 5C).

### 3.6 TGF‐β1 leads to enhanced levels of succinate

**FIGURE 6:**
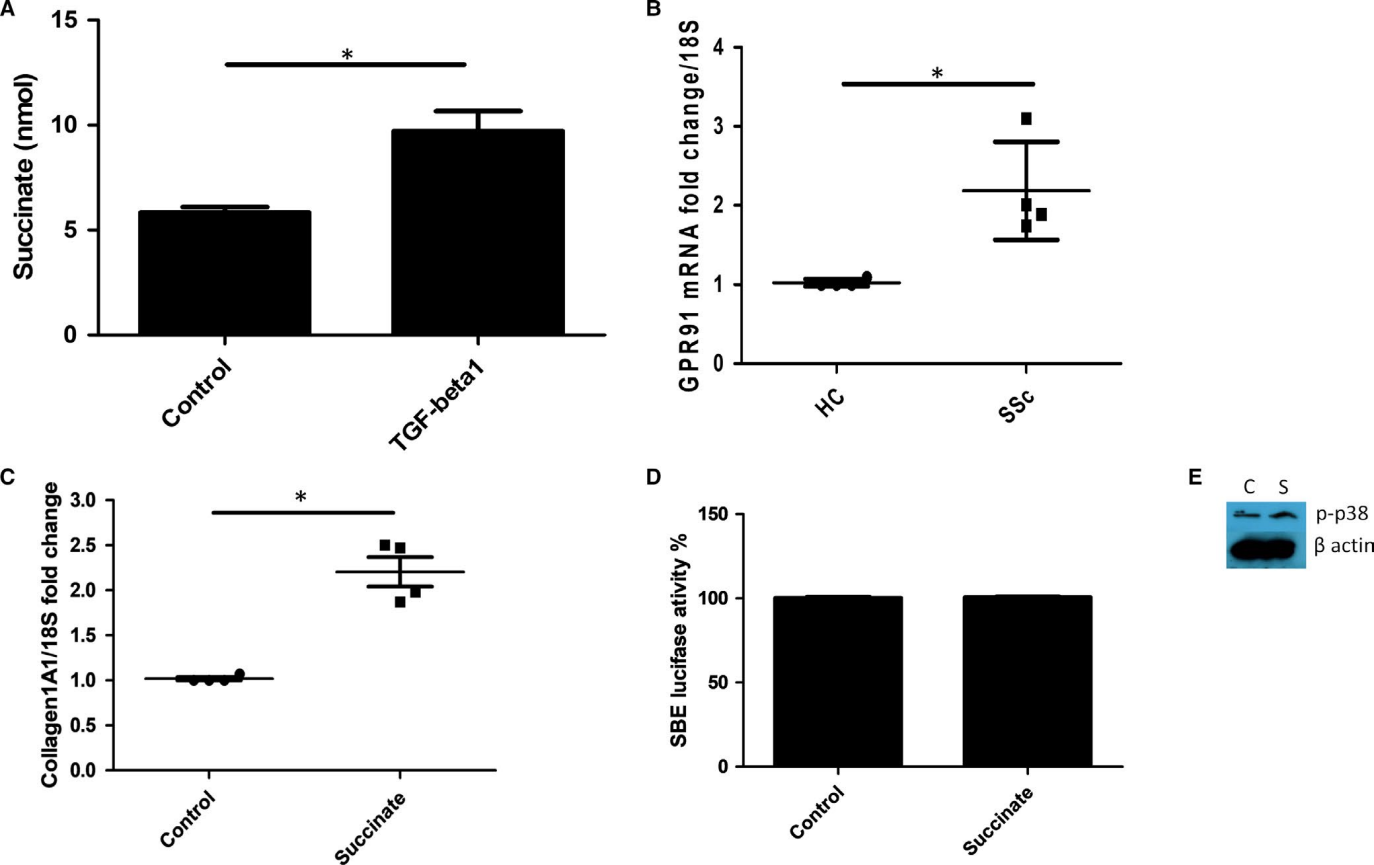
TGF‐β1 up‐regulates succinate levels. A, Healthy dermal fibroblasts were stimulated with TGF‐β1 or not, and after 36 h succinate was quantified colorimetrically * significantly different compared with controls *P* = .0002; Student's *t* test. Data are the mean and SD n = 4. B, GPR91 expression is higher in SSc dermal fibroblasts *Significantly different compared with healthy controls *P* = .0096 n = 4 donors. C, Collagen1A1 was quantified after succinate incubation (5 mmol/L) using qRT‐PCR, Data are normalized to the housekeeping gene 18S and shown as fold change compared with control. Data are mean and SD * significant difference compared with control *P* = .0005 Student's *t* test; n = 4. D, No significant difference in SMAD activation after succinate incubation. Luciferase activity is determined after stimulation with succinate. Data are normalized to untreated control set to 100% luciferase set to *Renilla* luciferase for transfection efficacy n = 3. E, Western blot of healthy dermal fibroblast incubated with succinate after 15 min and the cells were probed with phosphorylated p38 and re probed with β‐actin for equal loading. C = control untreated S = Succinate 5 mmol/L

Because of the production of α‐ketoglutarate after glutaminolysis, which can be enhanced by the pro‐fibrotic stimulus TGF‐β1, we postulated that the next product of the tricarboxycylic acid (TCA) cycle succinate would be elevated after TGF‐β1 stimulation. To this end, we stimulated isolated NHDFs with TGF‐β1 and found elevated levels of succinate 5.8 nmol SD 0.28 v 9.7 nmol SD 0.95, *P* = .0002 Student's *t* test n = 4 (Figure 6A).

Furthermore, we found elevated levels of the succinate receptor GPR91 in SSc dermal fibroblasts *P* = .0096 n = 4 (Figure 6B). Furthermore, treatment of healthy dermal fibroblasts with extracellular succinate (5 mmol/L) led to a significant increased collagen1A1 gene expression (Figure 6C) (1 vs 2.1 fold SD = 0.32; *P* = .0005 Student's *t* test; n = 4) and collagen release. Using a luciferase reporter for the Smad‐binding element, we could see no increase in luciferase activity after succinate incubation (Figure 6D, no significant difference control versus succinate treated Student's *t* test *P*=>.05; n = 3), indicating no TGF‐β activation. Because succinate in other systems has been found to activate the MAPK p38, we examined the phosphorylation status of p38 after stimulation with succinate (5 mmol/L) in normal healthy fibroblasts after 15 minutes. Figure 6E demonstrates the phosphorylation of p38 after 15 minutes by Western blot showing weak phosphorylation only (Figure 6E).

### 3.7 The metabolic modulator Itaconate reduces collagen

**FIGURE 7:**
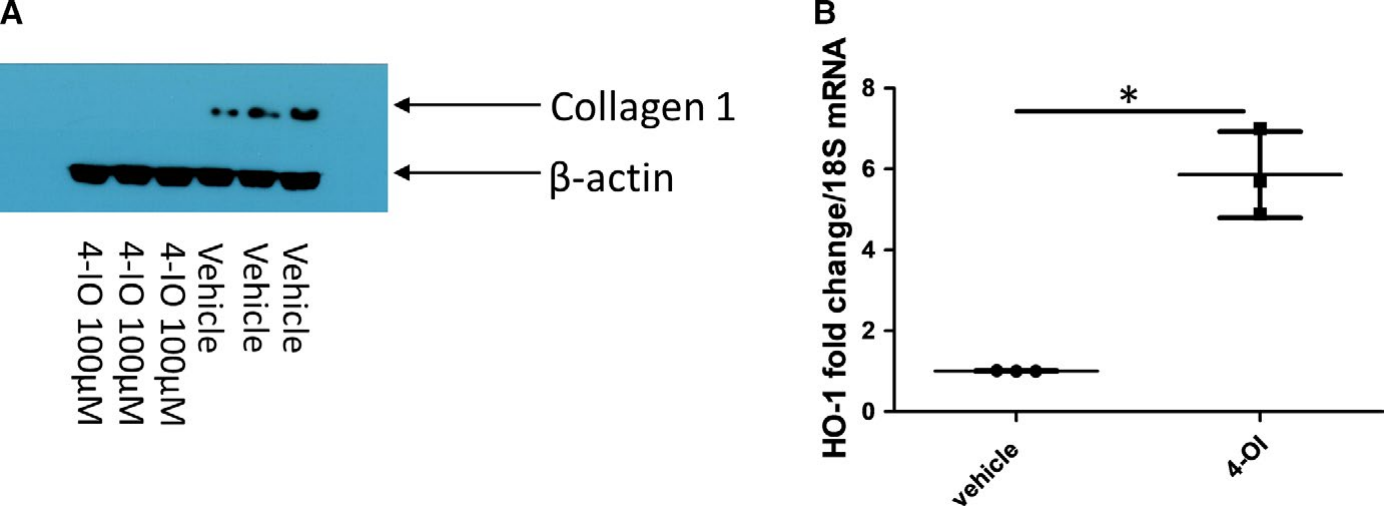
The metabolic regulator Itaconate reduces collagen in SSc fibroblasts. A, Western blot to quantify collagen 1 in SSc dermal fibroblasts exposed to the cell permeable 4‐octyl Itaconate (100 µmol/L) or vehicle control for 48 h. Cells were lysed and subjected to PAGE and transferred and probed with collagen 1 antibody. Β‐actin is used to confirm equal loading of protein. B, Elevated HO‐1 gene expression after Itaconate stimulation (100 µmol/L). Data are the mean from three individual donors each point a donor. Data are normalized to the housekeeping gene 18S (*P = .0014 Student's *t* test; n = 3)

Itaconate is metabolic by‐product of the TCA cycle. It is generated by the gene Immune‐responsive gene 1 (IRG1) by decarboxylating cis‐aconitate.^24^ This is one of the most highly up‐regulated molecules after inflammation is induced and inhibits oxidation of succinate and is potently anti‐inflammatory.^25^ The purpose of up‐regulation of itaconate appears to be to restrain inflammation and its subsequent consequences. Recently, a cell permeable derivative of itaconate termed 4‐octyl itaconate was derived and found to be both cell permeable and anti‐inflammatory by up‐regulating nrf‐2 and subsequent antioxidant genes.^21^ Thus, we used 4‐octyl itaconate in dermal fibroblast from three SSc donors. Figure 7A demonstrates that incubation with the cell permeable derivative of itaconate leads to reduced collagen in these cells compared with vehicle control. Nrf‐2 appears to be up‐regulated by itaconate in immune cells.^21^ Nrf‐2 is a potent master regulator of cellular stress response and sets in motion after up‐regulation a set of specific genes involved glutathione synthesis and antioxidant genes; one of which is Haem Oxygenase‐1 (HO‐1).^26^ HO‐1 gene expression is significantly elevated after itaconate treatment; vehicle mean fold change 1 (0.01) vs 5.86 (1.1) Figure 7B (*P* = .0014 Student's *t* test; n = 3). HO‐1 is a direct transcriptional target of the master antioxidant regulator nrf‐2 and therefore a proxy of nrf‐2 engagement.^26^

## 4 DISCUSSION

SSc and indeed other fibrotic conditions are characterized by excessive myofibroblast proliferation and the exuberant production of ECM.^27^ Resembling a milder version of the ‘Warburg effect’ observed in cancer,^28^ we show that the pro‐fibrotic stimulus TGF‐β1 up‐regulates glycolysis, with the concomitant inhibition of glycolysis preventing development of the fibrotic phenotype. We also find that succinate is elevated and that succinate itself up‐regulates collagen. We posit that the importance of glycolysis for TGF‐β1 driven fibrosis is in part due to the production of key biosynthetic intermediates for ECM synthesis and fibrosis. We also demonstrate that the importance metabolite itaconate reduces collagen in SSc fibroblasts.

However, in addition to glycolysis, the conversion of glutamine to glutamate is essential for the production of the key collagen I precursor α‐ketoglutarate and building blocks for non‐essential amino acid synthesis, suggesting glutamine metabolism is also integral for the development of fibrosis.^29^ This is evidenced by the ability of the specific glutaminase inhibitor G968 to attenuate collagen I expression in TGF‐β1 1 treated NHDFs. Interestingly, it did not reduce collagen at the transcript levels, suggesting a post‐transcriptional effect. Indeed, in liver fibrosis induced in animal models with carbon tetrachloride pre‐treatment with a glutaminase inhibitor potently reduced liver fibrosis.^30^ Indeed, liver fibrosis myofibroblasts, hepatic stellate cells, were highly dependent on glutaminolysis for their conversion and metabolic needs and inhibition with the compound we used, G968, potently reduced their fibrotic phenotype.^30^

Glutaminolysis has also been found to be critically important in mediating fibrosis in idiopathic lung fibroblasts ^31^ as siRNA‐mediated reduction of glutaminase 1 resulted in suppression of the production of collagen and other important ECM proteins.^31^ The mechanism in regard to the up‐regulation of anti‐apoptotic proteins associated with the pulmonary fibroblast phenotype involves histone trimethylation H3K27me3, which epigenetically regulates the target protein. Given that epigenetic regulation is also a key event in SSc^27^, ^32^ and in particular trimethylation has been shown to be important,^33^ we speculate that histone modification after production of α‐ketoglutarate may be critically important in ECM deposition. ROS has also been found to play a role in SSc^34^ and could also influence metabolism.

It was of note that there was significant variation in the levels of glycolysis, glycolytic capacity and glycolytic reserve between the SSc fibroblasts from different donors suggesting that the involvement of glycolysis may be highly variable between different patients and heavily influenced by a number of other factors. However, in line with its predicted role as a pro‐fibrotic pathway, glycolysis was elevated in the fibroblasts showing the highest collagen expression, although this is a very tentative correlation given the low sample size. This fits with the discovery that lactate was substantially elevated in SSc dermal blister fluids,^35^ which is in close contact to the underlying fibroblasts.

Unlike glycolysis, glutaminase 1 expression was elevated in all SSc fibroblasts, hinting that this may be a defining feature of the disease. Given TGF‐β1 increases glycolysis and is known to be elevated in SSc, it may be that the combination of TGF‐β1‐enhanced glycolysis and endogenous up‐regulation of glutamine metabolism is key to the development of fibrotic skin lesions in SSc.

We further show that TGF‐β1 leads to up‐regulation of the key enzyme that mediate glutaminolysis in normal dermal fibroblasts and that this is mediated, at least partly, through Smad activation. The cellular use of glutamine begins with the enzyme glutaminase that initiates glutaminolysis to generate glutamate and subsequently α‐ketoglutarate via glutamine dehydrogenases.^36^ Although the induction of glutaminase 1 could also be partially mediated through non‐canonical TGF‐β signalling pathways. To examine the role of Smad‐independent pathways after TGF‐β1 stimulation of glutaminase 1, we inhibited the FGFR3 and demonstrated that this inhibition also attenuated glutaminase 1 induction. Of note, it was recently described that FGFR3 inhibition reduced lung fibrosis markers in isolated lung fibroblasts mediated by TGF‐β and in the bleomycin lung fibrosis model FGFR3 inhibitors reduced fibrosis in vivo^37^ and its ligand FGF9 is found in hepatic stellate cells of the fibrotic liver.^38^ In a recent manuscript, it was demonstrated that in lung fibroblasts TGF‐β1 leads to up‐regulation of glutaminase 1 in these cells via Smad‐dependant pathways and non‐canonical pathways.^39^ Indeed, it was demonstrated that siRNA‐mediated reduction in Smad3 reversed the up‐regulation of glutaminase 1 and that the effect was also mediated by down‐regulation of the NAD + deacetylase Sirtuin 7 (SIRT7) leading to elevated acetylation of FOXO1 that is then able to directly bind the promoter of glutaminase 1^39^—as acetylation is more permissive to transcription. Although we did not look at the levels of SIRT7 after TGF‐β1 stimulation it could be that as in the lung,^39^ this leads to reduced deacetylation of FOXO1 and binding of the promoter. It is interesting to note, however, that SIRT7 has been found to be significantly decreased in the skin and lungs of SSc patients^40^ and that enforced overexpression of SIRT7 through a SIRT7‐GFP plasmid reduced collagen and α‐smooth muscle actin levels.^40^ Although this requires further experimentation, this could be the underlying mechanism in the increase in glutaminase 1.

Furthermore, we show that stimulation of NHDFs with TGF‐β1 leads to elevated succinate levels. Succinate is a metabolite of the TCA cycle that has recently been identified as not merely a by‐product but an active metabolite, in some cases acting as a danger signal.^41^, ^42^ Indeed, succinate has been found to enhance immunity and signal through a G protein‐coupled receptor (GPR91)^43^ and exacerbates a mouse model of arthritis.^44^ Mice which are knocked out for GPR91 have reduced immune rejection to transplant.^43^ Succinate appears to be a danger signal that leads to activation of hepatic stellate cells, the myofibroblasts of the liver.^45^

Furthermore, succinate infusion has been found to cause cardiac fibrosis in a GPR91‐dependant fashion and stimulates ERK phosphorylation.^46^ Recently, it was demonstrated that succinate was associated with fibrosis in inflammatory bowel disease and these patients have higher levels of GPR91 expression.^47^ Additionally, mice with GPR91 deletion were protected from fibrosis in the intestine and GPR91 KO isolated fibroblasts could not up‐regulate collagen after succinate incubation.^47^ We found significantly higher levels of the succinate receptor GPR91 in SSc cells and addition of extracellular succinate led to elevated collagen expression in dermal fibroblasts. This suggests that succinate itself is pro‐fibrotic. Of course, succinate is made in a variety of cell types and in SSc succinate could be released from macrophages to active the fibroblast to undergo fibrotic changes leading to enhanced ECM. Given that macrophages are key cell types in SSc,^48^, ^49^ this is a plausible explanation. How succinate may up‐regulate collagen is unknown but appears to be independent of TGF‐β1 as the Smad reporter assay did not increase after succinate incubation. Others have demonstrated succinate mediates its effects through stabilization of Hypoxia Inducible factor 1‐α (HIF‐1α).^50^ It is known that HIF‐1α is a pro‐fibrotic transcription factor. We also saw some weak phosphorylation of p38, as others have suggested that succinate can signal through this intermediate. Although it is unknown, whether this weak activation of P38 is sufficient for ECM deposition.

We also demonstrate that 4‐octyl itaconate—a cell permeable form of itaconate reduced collagen1 expression in SSc dermal fibroblasts. Itaconate is a by‐product of the TCA cycle that is hugely up‐regulated in inflammation.^51^ Although associated with activated immune cells, itaconate is found in many stromal cell compartments also^51^ indicating it is not restricted to immune cells exclusively. Thus, it appears that itaconate is anti‐inflammatory presumably up‐regulated to restrain unwanted and damaging persistent inflammation. The mechanism has recently been shown to be through up‐regulation of nrf‐2, via alkylation of specific residues on keap1.^21^ Keap1 is the negative regulator of nrf‐2 by targeting this for ubiquitination and subsequent cellular degradation^26^—thereby regulating tight control of its activity. Recent studies have shown that modifications to keap1 by a derivative of itaconate lead to enhancement of nrf2 and downstream reduced inflammation, in a model of skin inflammation‐driven by IL‐17—itaconate reduced this skin pathology^25^ significantly. We show here that itaconate reduced collagen significantly and that the bona fide target gene of nrf‐2, HO‐1 is significantly up‐regulated in these cells. In a recent study, itaconate and its derivative were shown to reduce liver damage and fibrosis in a ischaemia reperfusion model of liver injury.^52^ Furthermore, it was recently shown that itaconate reduces inflammation through inhibition of glycolysis metabolism via covalent modification of GAPDH.^53^ Interestingly, mice in whom nrf2 is reduced are less protected from the effects of itaconate—we suggest that itaconate is anti‐fibrotic and is dependent upon nrf2 for this action. Interestingly, a previous study has demonstrated significantly reduced nrf2 expression in skin and lung tissues from SSc patients and nrf2 knockout mice has much exaggerated skin fibrosis in the bleomycin mouse model.^54^ Furthermore, incubation of cells with interferon‐β significantly enhanced the concentration of intracellular itaconate,^21^ which may explain the reduction of collagen and alpha‐smooth muscle actin in SSc fibroblasts by interferon‐β.^55^

**FIGURE 8:**
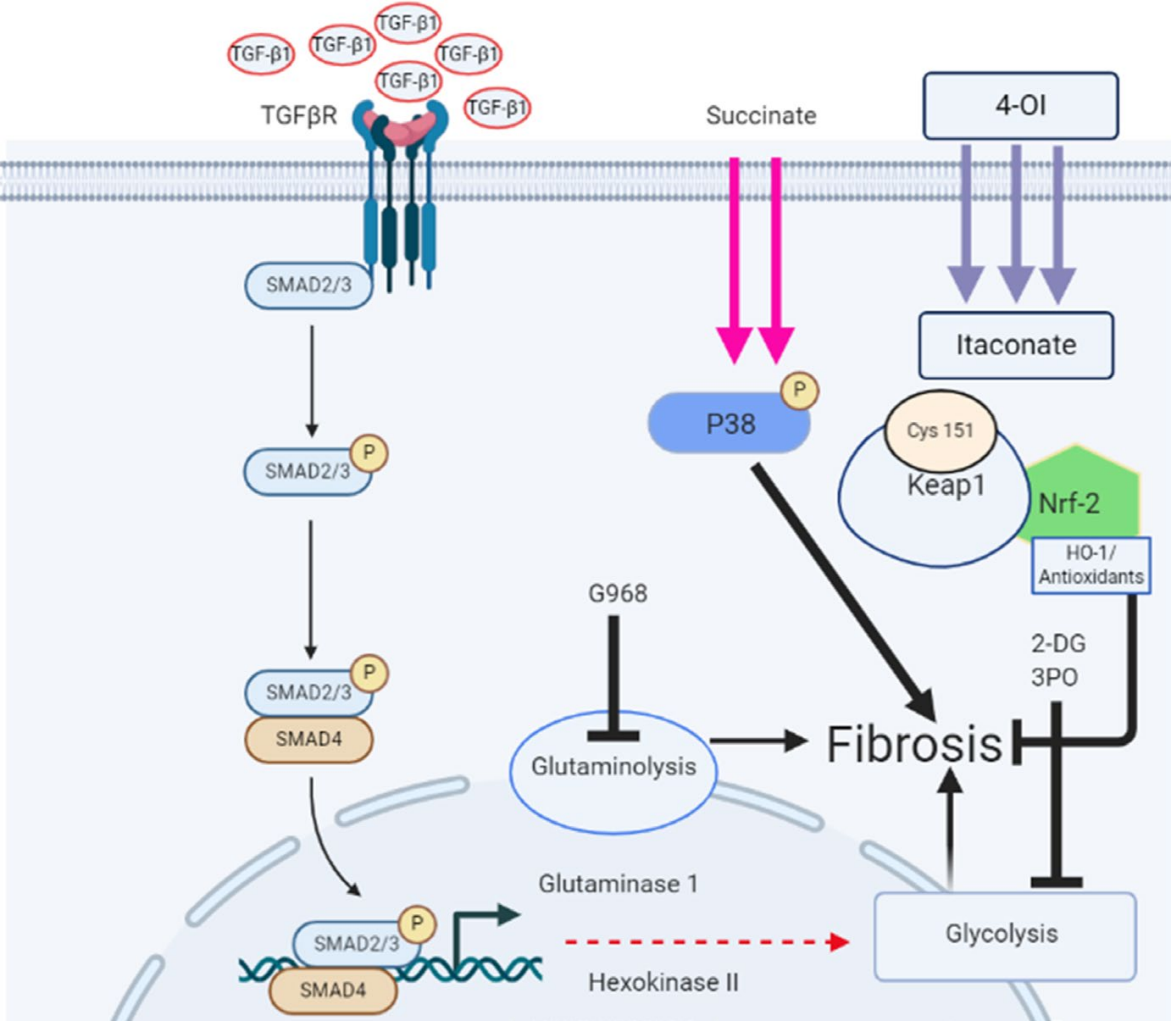
Putative pathway of metabolic alterations via TGF‐β1 TGF‐β1 binds the cognate receptor and then causes downstream signalling from the canonical pathway to activate glutaminolysis via up‐regulation of glutaminase 1 enzyme, and this leads to fibrosis possibly through increased epigenetic marks. Glycolysis is also up‐regulated leading to fibrosis via up‐regulation of glycolytic enzymes. Both pathways can be blocked with G968 (glutaminase 1 inhibitor) or 2‐DG and 3PO for glycolysis. Extracellular succinate also up‐regulates fibrosis possibly through the activation of P38 via phosphorylation of this intracellular signalling molecule. 4‐OI mediates an anti‐fibrotic effect by first being cleaved to itaconate via esterases and alkylates cysteine residue 161 (Cys 161) of KEAP1 thus repressing its ability to modify nrf‐2 to be degraded via the proteosome. Nrf‐2 is then up‐regulated and downstream targets such as HO‐1 are increased that are anti‐fibrotic. 2‐DG; 2Deoxyglucose, 4‐OI; 4‐octyl itaconate, HO‐1; Haem oxygenase‐1, nrf2; nuclear factor erythroid‐2‐related factor, TGF‐β1; transforming growth factor beta 1

Metabolism and metabolic reprogramming have recently seen a renaissance in research, and its importance is now being appreciated in many diseases.^56^ Importantly, this introduces the possibility of applying metabolically targeted interventions such as 3PO and G968 for the treatment of SSc. Furthermore, the novel observation of succinate dysregulation in SSc warrants further investigation. Finally, we show that the important metabolic product itaconate can reduce collagen in SSc fibroblasts. Figure 8 illustrates a putative pathway where inhibition can occur and the use of itaconate and its possible mechanism. Given that itaconate has now been shown to inhibit the inflammasome and reduce severity in an animal model of peritonitis^57^ and liver fibrosis,^52^ it may be considered in skin fibrosis.

## CONFLICT OF INTEREST

The authors state there are no conflicts of interest to declare.

## AUTHOR CONTRIBUTION

**John Henderson:** Data curation (equal); Formal analysis (equal); Investigation (equal); Writing‐review & editing (equal). **Laura Duffy:** Methodology (supporting). **Richard Stratton:** Investigation (equal); Methodology (equal); Writing‐review & editing (equal). **Dianne Ford:** Data curation (equal); Formal analysis (equal); Funding acquisition (equal); Supervision (equal); Writing‐review & editing (equal). **Steven O'Reilly:** Conceptualization (lead); Formal analysis (lead); Methodology (lead); Resources (equal); Supervision (equal).

## Supporting information

Fig S1
