# Supplementary figures and images for: Metabolic reprogramming of glycolysis and glutamine metabolism are key events in myofibroblast transition in systemic sclerosis pathogenesis
Source: J Cell Mol Med. 2020 Nov 2;24(23):14026–38. doi: 10.1111/jcmm.16013 (PMC7754020; doi:10.1111/jcmm.16013)

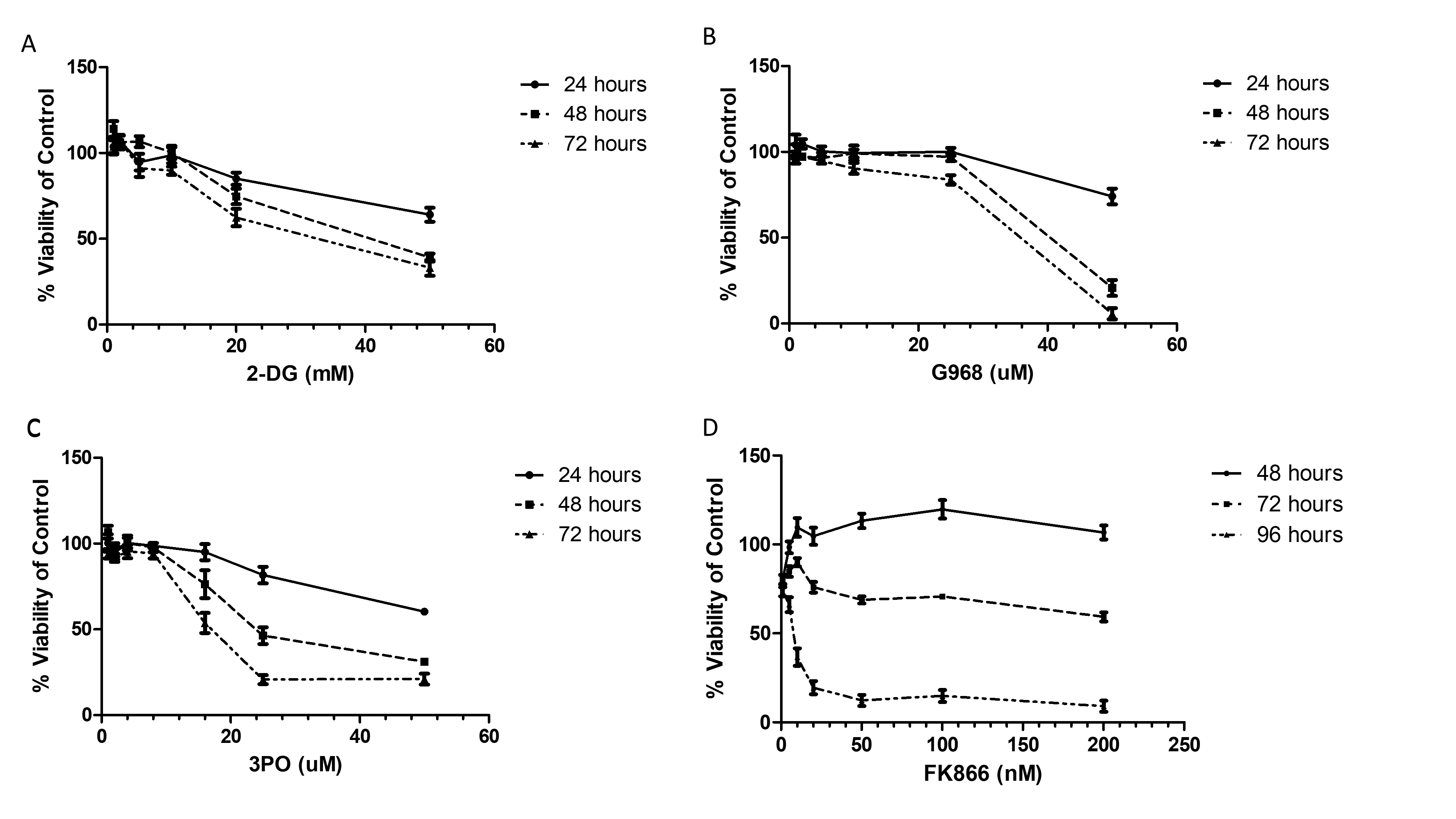

Supplement: Supplementary file 1 — Fig S1 [file JCMM-24-14026-s001.tif]
